# Reduced bacillary load in elderly patients with active extrapulmonary and pulmonary tuberculosis in Peru: analysis of confirmatory culture after acid-fast bacilli test

**DOI:** 10.3389/fmicb.2024.1398999

**Published:** 2024-11-14

**Authors:** Jeel Moya-Salazar, Jonathan Samán, Israel A. Pasco, Marcia M. Moya-Salazar, Víctor Rojas-Zumaran, Hans Contreras-Pulache

**Affiliations:** ^1^Vicerrectorado de Investigación, Universidad Norbert Wiener, Lima, Peru; ^2^CENEX, Hospital Nacional Hipólito Unanue, Lima, Peru; ^3^Department of Laboratory Medicine, Centro de Salud Hermitaño Bajo, Lima, Peru; ^4^Infectious Unit, Nesh Hubbs, Lima, Peru; ^5^Department of Pathology, Hospital Nacional Docente Madre-Niño San Bartolomé, Lima, Peru

**Keywords:** tuberculosis, aged, Ogawa, smear, extrapulmonary tuberculosis, Peru

## Abstract

**Background:**

Older adults with tuberculosis (TB) present unusual clinical features and can be challenging to diagnose. Culture after evaluation of sputum smear (AFB) may result in improved diagnosis performance, however it has not yet been evaluated in Peruvian older adults. We aimed to evaluate the diagnostic relation of TB culture after the AFB in patients aged ≥ 65 years derived for the diagnosis of pulmonary (PTB) and extra-pulmonary (EPTB) in Lima, Peru.

**Methods:**

A cross-sectional study was developed in Lima, Peru, in order to evaluate the relationship of TB culture after AFB test in older adults (≥ 65 years) during the PTB and EPTB diagnosis. The frequency of contaminated cultures and the discrepancies between the conventional AFB test and Ogawa-Kudoh culture were analyzed.

**Results:**

Of the 10,461 sputum and 2,536 extrapulmonary samples analyzed during 2015–2017, PTB was diagnosed in 282 (2.7%) and EPTB in 88 (3.5%), respectively. The performance of AFB in the diagnosis of PTB had a sensitivity of 78.2% and specificity of 99.8%. The performance of AFB in EPTB had a sensitivity of 45.5% and specificity of 99.9%. Negative AFB with positive culture was more frequent in ≥ 82 years (*p* = 0.031). We determined a good agreement in the diagnosis of PTB (κ = 0.84) and moderate for EPTB (κ = 0.55).

**Conclusion:**

Our findings suggest that diagnosis through culture should be performed after the AFB smear evaluation due to the moderate performance of AFB, especially in patients ≥ 82 years old.

## 1 Introduction

For many centuries, tuberculosis (TB) has caused significant economic and social impacts worldwide, particularly in low-income countries where it remains one of the leading causes of mortality (World Health Organization, 2022). TB is a complex issue that requires the combined efforts of governments, the private sector, and civil society.

Peru is a middle-income country−with more than 33 million inhabitants - that has a TB incidence of 87.6 per 100,000 inhabitants in 2017 (Alarcón et al., 2017). Although the population is constantly exposed to this disease, there are risk groups such as prisoners, healthcare workers, patients with type 2 diabetes, and the elderly where the disease worsens (Rojas-Bolivar et al., 2018; Ugarte-Gil et al., 2021). Pulmonary TB (PTB) accounts for more than 80% of cases, with diagnosis primarily conducted through acid-fast bacilli (AFB) smear analysis and bacteriological culture (Ministerio de Salud, 2006, 2023). Given the high incidence of this disease, it is crucial to prioritize accurate and rapid diagnostic methods that align with the community’s reality (Pan-American Health Organization, 2013).

In Peru, it is estimated that there are around 4 million elderly people. The most common diseases in this population include hypertension, diabetes, and cardiovascular diseases (Instituto Nacional de Estadística e Informática, 2020, 2023). TB also affects the elderly, with a rate of approximately 120 cases per 100,000 people, and 11–20% of these cases being new each year (Alarcón et al., 2017; Ministerio de Salud, 2024). Diagnostic complications have been observed in elderly patients with TB that could interfere with the current diagnostic approach in Peru, focused on AFB smear analysis (Ministerio de Salud, 2018). The elderly population presents a different clinical profile with fewer evident symptoms, a distinct immune response to TB, and complex reactions to treatment due to physiological changes associated with aging. These factors pose challenges for diagnostic tests (Teo et al., 2023; Sharma et al., 2022; Yoshikawa and Rajagopalan, 2001), potentially leading to a lack of confirmed diagnoses in these patients.

The absence of culture confirmation in the majority of AFB-based studies hinders the ability to estimate test performance differences, which may affect diagnostic quality. Cross-contamination of cultures is a common error, estimated at 2% of all positive TB cultures, resulting in 9.2% of patients receiving false positive results and inadequate treatment (Barac et al., 2019). Additionally, previous reports have emphasized the importance of laboratory performance for PTB diagnosis (Symes et al., 2020; Silva et al., 2013; Cruz-Hervert et al., 2012; Rivas et al., 2010; Yoshikawa and Rajagopalan, 2001). However, the impact of diagnostic tests on extrapulmonary TB (EPTB) samples has not been thoroughly evaluated.

In the context of global aging, the elderly have a high mortality rate, poor clinical clarity in TB symptoms, and face limitations with current diagnostic methods. Thus, the diagnostic performance of AFB smear and culture should be carefully evaluated (Sood, 2000; Partridge et al., 2018; Teo et al., 2023; Sharma et al., 2022). Improving TB diagnostic programs for the elderly is essential, especially in countries with high TB prevalence and complex social, demographic, health access, and economic conditions (Purohit and Mustafa, 2015; Colegio Médico del Perú, 2021; Huarachi et al., 2024).

We aimed to evaluate the diagnostic relation of TB culture after the evaluation of sputum smear (AFB) in older patients derived for the diagnosis of PTB and EPTB in Lima, Peru. We also determined the frequency of contaminated results by sex, age group, and treatment history.

## 2 Materials and methods

### 2.1 Study design and sample

This cross-sectional study was conducted at the Hipólito Unanue National Hospital in Lima, Peru. The hospital is located in the Lima downtown, in the district of El Agustino (12°4′0″S, 77°1′0″W), one of the districts with a high incidence of TB, high poverty rates, and high population density (15,416.19 inhabitants/km^2^) (Instituto Nacional de Estadística e Informática, 2015). The patients included in this study were all aged 65 years or older and resided in this district. All patients attending the TB Diagnostic Service had been referred by their treating physician after consultation to be included in the TB Control Program (PCT). This program has nationwide coverage to promote the prevention and control of TB, ensuring consistent diagnosis and treatment for both TB and non-TB patients.

We included patients over 65 years of age from both genders, from the PCT and outpatient clinics of the hospital, regardless of previous TB treatment history. Both pulmonary and extrapulmonary samples (i.e., cerebrospinal fluid) were included. Patients with extremely drug-resistant TB (XDR-TB) or multidrug-resistant TB (MDR-TB) were excluded to avoid bias in the interpretation of AFB smear and culture results. The clinical diagnosis for PTB involved identifying respiratory symptoms (i.e., cough) or a recent positive PPD test, abnormal chest X-ray findings, positive AFB smear, and initiating or controlling treatment according to the Peruvian treatment regimen (Ministerio de Salud, 2023).

### 2.2 TB diagnostic methods

All sputum and extrapulmonary samples were processed by the Microbiology Area team and the Research Department for TB diagnosis and treatment, following the hospital’s bacteriological guidelines (Sequeira de Latini and Barrera, 2008; Barrera, 2008). The conventional AFB sputum test was performed by preparing a slide with the patient’s sample and staining it with Ziehl-Neelsen following national guidelines (Ministerio de Salud, 2023). Cultures were performed on Ogawa-Kudoh agar (Merck, Darmstadt, Germany) in accordance with international quality standards (Rivas et al., 2010; Sequeira de Latini and Barrera, 2008; Barrera, 2008; Asencios et al., 2012; Clinical and Laboratory Standards Institute, 2008). Extrapulmonary specimens, such as gastric aspirate, feces, urine, and pleural fluid, were collected by hospital departments teams (i.e., gastroenterology) and promptly sent for laboratory analysis. These samples were also processed using smear microscopy and cultured on Ogawa-Kudoh agar.

As previously reported (Moya-Salazar et al., 2018), the evaluation of samples adheres to national quality regulations (Ministerio de Salud, 2020, 2023). This ensures that processes are continuously monitored under protocols established by the National Institute of Health and the Ministry of Health of Peru (Asencios et al., 2012). By following these diagnostic and result interpretation guidelines, we minimize interpretation bias in AFB smears and acknowledge the limitations of culture results.

### 2.3 Confirmatory culture evaluation and data analysis

We analyzed all patients with discordant or concordant AFB and culture results (primarily AFB-negative with positive culture) and evaluated the frequency of contaminated results. To establish the relationship between results, they were categorized into the following groups:

1. Sputum smear (AFB): paucibacillary results (AFB per 100x fields), one cross 1 (+), two crosses 2 (++), and three crosses 3 (+++).
2. Culture: number of colonies grown in the medium (No. 1 to 19 colonies), one cross 1 (+), two crosses 2 (++), and three crosses 3 (+++).

All these results (PTB and EPTB) were coded from the hospital’s data storage system (MS-Excel 2010, Redmond, US) into the data matrix (EPIDAT v4.1, Xunta de Galicia, Spain). We used descriptive statistics and non-parametric correlation between study variables, considering a 95% confidence interval (CI 95%) and a *p*-value < 0.05 as statistically significant. To determine the performance of the AFB smear, diagnostic tests (sensitivity, specificity, Positive Predictive Value (PPV), Negative Predictive Value (NPV), accuracy, and Youden’s Index) and the Receiver Operating Characteristic (ROC) curve were used. Statistical analysis was performed using the Statistical Package for the Social Sciences (SPSS) v25.0 (IBM, Armonk, US) and EPIDAT v4.1 for Windows.

### 2.4 Ethical aspects

We follow the Helsinki declaration guidelines (World Medical Association, 2013) and this study was approved by the IRB of the Universidad Norbert Wiener (Exp. N° 103-2017-UNW, March 12, 2017).

## 3 Results

### 3.1 Descriptive results

Out of 31,819 patients, we enrolled 12,997 (45%) who were older than 65 years. The distribution by year was as follows: 4,136 (31.8%) patients in 2015, 4,511 (34.7%) in 2016, and 4,350 (33.5%) in 2017. The average age was 75 ± 7.5 years (CI 95%, 75 to 75.3 years). Of these, 6,499 (50.1%) were women, with no significant age difference between genders (*p* = 0.101). Five patients (0.03%) had no recorded sex. We registered 10,357 (79.7%) patients without previous treatment, 1,942 (14.9%) with previous treatment, and 698 (5.4%) without registration (Table 1).

**TABLE 1:** Baseline characteristics of elderly patients (≥ 65 years) with extra-pulmonary and pulmonary tuberculosis. Data in *n* (%).

Characteristics		Tuberculosis¶						
		Normal		PTB*		EPTB*		Total
		61–82	≥ 82	61–82	≥ 82	61–82	≥ 82	
***Study period* ^†^**								
	2015	3104 (24)	846 (6.5)	76 (0.57)	33 (0.25)	26 (0.2)	11 (0.08)	4096 (31.5)
	2016	3402 (26)	955 (7.3)	84 (0.65)	10 (0.08)	11 (0.08)	10 (0.08)	4472 (34.4)
	2017	3314 (25)	896 (6.9)	64 (0.45)	15 (0.12)	19 (0.15)	11 (0.08)	4319 (33.2)
***Gender***								
	Women	4897 (37.7)	1472 (11.3)	88 (0.7)	23 (0.18)	18 (0.14)	16 (0.12)	6514 (50.12)
	Men	5014 (38.6)	1243 (9.6)	134 (1.03)	39 (0.3)	23 (0.18)	30 (0.23)	6483 (49.88)
***Treatment***								
	BT^‡^	1580 (12.2)	270 (1.6)	65 (0.5)	11 (0.08)	9 (0.07)	1 (0.008)	1936 (14.9)
	NT	7819 (60.2)	2300 (17.7)	149 (1.15)	37 (0.28)	39 (0.3)	29 (0.22)	10373 (79.8)
	NR	503 (3.8)	158 (1.22)	17 (0.13)	0 (0)	8 (0.06)	2 (0.015)	688 (5.3)
***Types of samples***								
	Sputum	8117 (62.5)	2054 (15.8)	234 (1.8)	48 (0.36)	…**	…	10453 (80.4)
	Gastric aspirate	457 (3.5)	310 (2.4)	…	…	16 (0.2)	15 (0.11)	798 (6.14)
	Pleura liquid	300 (2.3)	111 (0.85)	…	…	3 (0.023)	2 (0.015)	416 (3.2)
	Bronchial aspirate	383 (2.9)	117 (0.9)	…	…	18 (0.13)	9 (0.06)	527 (4)
	Post. Bronchial aspirate	130 (1)	9 (0.06)	…	…	5 (0.03)	3 (0.023)	147 (1.13)
	Urine	180 (1.38)	33 (0.25)	…	…	4 (0.03)	0 (0)	217 (1.7)
	Stool	76 (0.6)	16 (0.12)	…	…	1 (0.008)	0 (0)	90 (0.7)
	Tracheal aspirate	64 (0.5)	16 (0.12)	…	…	3 (0.023)	2 (0.015)	85 (0.6)
	Cerebrospinal fluid	52 (0.4)	18 (0.13)	…	…	0 (0)	0 (0)	70 (0.5)
	Pericardium biopsy	10 (0.08)	0 (0)	…	…	0 (0)	0 (0)	10 (0.08)
	Cervical abscess	9 (0.06)	1 (0.008)	…	…	0 (0)	0 (0)	10 (0.08)
	Endotracheal tube	8 (0.06)	0 (0)	…	…	0 (0)	0 (0)	8 (0.06)
	Pericardial fluid	7 (0.05)	1 (0.008)	…	…	0 (0)	0 (0)	1 (0.008)
	Others	110 (0.8)	25 (0.2)	…	…	6 (0.05)	1 (0.008)	32 (0.24)

^¶^All patients were subdivided into two age groups: first group of 65–71 years, and second of ≥ 82 years.

*Diagnosis with both AFB test and culture test are included.

^†^The total of contaminated cultures pear year were excluded: 2015 (*n* = 40), 2016 (*n* = 39), and 2017 (*n* = 31).

**Includes patients with previous diagnosis and treatment control. No samples are recorded. EPTB, Extra-pulmonary tuberculosis; PTB, Pulmonary tuberculosis; NT, Never treated; NR, No report.

Out of the 10,461 sputum samples analyzed, TB was diagnosed in 282 (2.7%) samples from patients aged ≥ 65 years with and without symptoms. In 2015, 3,311 (31.7%) sputum samples were evaluated, with 109 (1%) positive and 37 (0.4%) showing culture contamination. In 2016, 3,666 (35%) samples were evaluated, with 94 (0.9%) positive and 28 (2.5%) contaminated. In 2017, 3,484 (33.3%) sputum samples were evaluated, with 79 (0.8%) positive and 17 (0.2%) contaminated in the Ogawa culture.

Of the 2 536 extrapulmonary samples that were analyzed, TB was diagnosed in 88 (3.5%) samples from patients ≥ 65 years with and without symptoms. In 2015, 824 (32.5%) sputum samples were evaluated, with 37 (1.5%) testing positive and 40 (1.6%) showing culture contamination. In 2016, 845 (33.3%) samples were evaluated, with 21 (0.8%) testing positive and 39 (1.5%) contaminated. In 2017, 867 (34.2%) sputum samples were evaluated, 30 (1.2%) were positive and 31 (1.22%) were contaminated in the Ogawa culture.

A very good diagnostic agreement between AFB test and culture was determined for the diagnosis of PTB (κ = 0.84, CI 95%, 0.79 to 0.89%) and moderate agreement for EPTB (κ = 0.55, CI 95%, 0.25 to 0.86%).

### 3.2 Diagnostic performance of PTB

The diagnostic agreement of positive results in pulmonary samples was evidenced in 1.1% of sputum smears (111/10461). The average age of these patients was 70.5 ± 6 (CI 95%, 69.3 to 71.7), where 45 were women and the history of previous treatment was not significant (*p* = 0.0334). We found that 0.05% of samples (5/10,461) presented positive sputum smears and negative culture results; all these samples were from women without prior treatment, with an average age of 72 ± 7.1 years. Additionally, 0.1% (12/10,461) of sputum smears were negative, but culture-positive. Five of these samples were from women (72.2 ± 6.5 years, CI 95%, 68.5 to 75.8), eight samples came from the Emergency Services, and only two had previous treatment.

**FIGURE 1:**
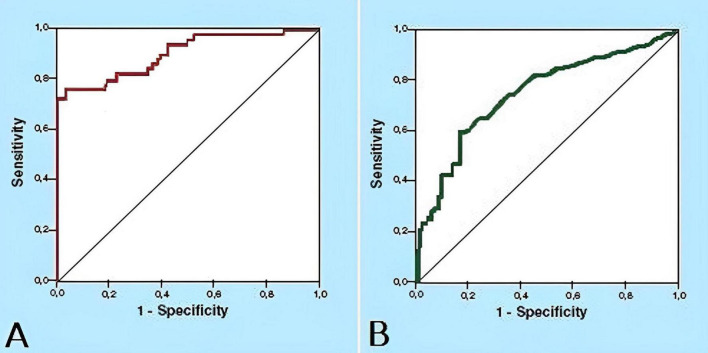
Receiver operating characteristic (ROC) curves for AFB test on the diagnosis of pulmonary and extrapulmonary tuberculosis in elderly patients (≥ 65 years) in Lima, Peru. **(A)** ROC curve for AFB test on the diagnosis of pulmonary tuberculosis. **(B)** ROC curve for AFB test on the diagnosis of extrapulmonary tuberculosis.

In the sputum analysis, the proportion of false positives was 0.2% (CI 95%, 0.1 to 0.3), and the proportion of false negatives was 21.8% (CI 95%, 15.8 to 29.3). From the analysis of the AFB tests in PTB diagnosis we obtained a sensitivity of 78.2% (CI 95%, 70.7 to 84.2), a specificity of 99.8% (CI 95%, 99.7 to 99.9), a PPV of 91% (CI 95%, 84.6 to 94.9), to NPV of 99.6% (CI 95%, 99.4 to 99.7), an accuracy of 99.4% (CI 95%, 99.2 to 99.6), and a Youden’s Index of 0.8 (Figure 1A).

### 3.3 Diagnostic performance of EPTB

Concerning extrapulmonary samples, diagnostic agreement of positive results was evidenced in 0.24% of samples (12/2,536). The average age of these patients was 76.2 ± 7 years (CI 95%, 70.1 to 82.3), with none having a history of treatment, and all samples being bronchial aspirates. Additionally, 0.07% (2/2,536) of samples had positive AFB but negative culture results. Conversely, 0.25% (6/2,536) of samples showed negative sputum smears but positive culture results. All these samples were bronchial aspirates, and one was from a woman (72 ± 5.7 years, CI 95%, 67 to 77).

The analysis of extrapulmonary samples allowed us to estimate a false positive rate of 0.1% (CI 95%, 0.0% to 0.3%) and a false negative rate of 54.5% (CI 95%, 28% to 78.7%). The analysis of extrapulmonary samples allowed us to estimate a false positive rate of 0.1% (CI 95%, 0.0% to 0.3%) and a false negative rate of 54.5% (CI 95%, 28% to 78.7%). From the analysis of the AFB tests in EPTB diagnosis, we obtained a sensitivity of 45.5% (CI 95%, 21.3 to 72), a specificity of 99.9% (CI 95%, 99.7 to 100), a PPV of 71.4% (CI 95%, 35.9 to 91.8), an NPV of 99.8% (CI 95%, 99.5 to 99.9), an accuracy of 99.7% (CI 95%, 99.4 to 99.8), and a Youden’s Index of 0.5 (Figure 1B).

### 3.4 Contamination frequency

**FIGURE 2:**
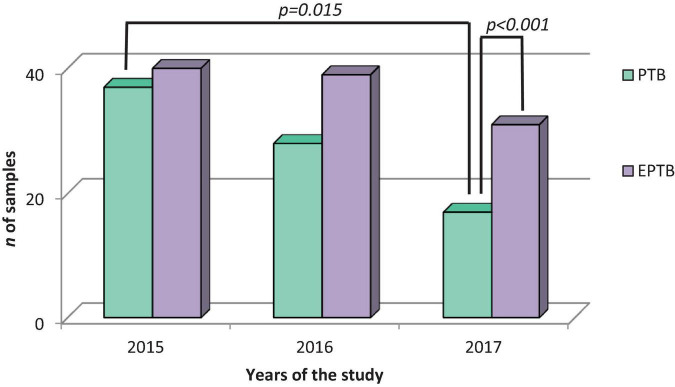
Distribution of contaminated cultures of pulmonary and extrapulmonary tuberculosis samples of elderly patients. During the study period shows the significant difference between the samples with contaminated results between 2015 and 2017 and the difference between sputum and extrapulmonary samples of the year 2017 (*n* = 161).

Regarding the results with contaminated culture, 40 (14.2%) sputum samples presented a contaminated result in 2015, one of which had a positive AFB result (2 + +) and a history of previous treatment. In 2016 and 2017 there were 39 (13.8%) and 31 (11%) sputum samples with contaminated culture, respectively. In the diagnosis of EPTB, 82 (29.1%) cases were recorded in total, with 12 (4.3%) contaminated cultures having positive sputum smears. No correlation was found between the contaminated cultures and the age of the patients (p = 0.573), nor with the history of previous treatment (*p* = 0.415) (Figure 2).

### 3.4 Performance analysis by age group

**FIGURE 3:**
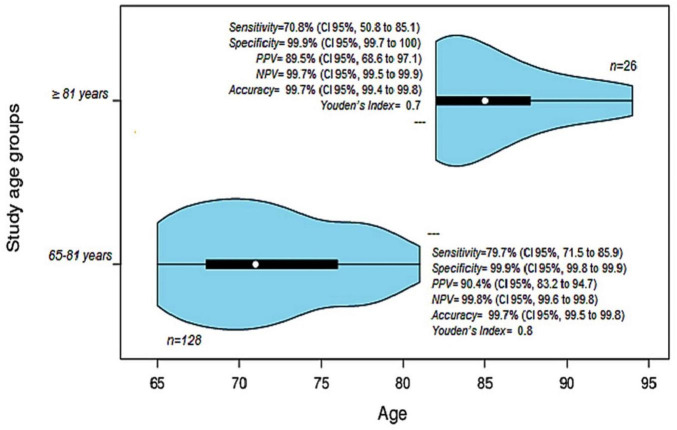
Diagnostic tests and analysis of the performance of the AFB test for the diagnosis of PTB in two age groups. The first group of 65–81 years (71 ± 5.2 years, CI95% 71 to 76) is shown in the violin below. The second group ≥ 82 years (85 ± 7.8 years, CI95% 82 to 87.75) is shown in the violin above. PPV, Positive predictive value, NPV, Negative predictive value.

The performance analysis of the AFB test was conducted in two age groups (65–81 years and ≥ 82 years) for the diagnosis of PTB and EPTB. In the first group (65–81 years), it was observed that 0.24% (24/10,201) presented AFB-negative results with a positive culture, and 0.1% (10/10,201) presented AFB-positive results with a negative culture. In the second group (≥ 82 years), 0.25% (7/2,796) had AFB-negative results with a positive culture, and 0.07% (2/2,796) had AFB-positive results with a negative culture. Negative sputum smears were more frequent in the ≥ 82 years group (*p* = 0.031), resulting in a false-positive rate of 0.1% (CI 95%, 0.1 to 0.2) and a false-negative rate of 20.3% (CI 95%, 14.1 to 28.5). Additional data is shown in Figure 3.

For the diagnosis of EPTB, the same analysis could not be performed due to the limited number of viable samples. However, AFB-negative with positive culture results were observed only in the first group (6/13). In patients aged ≥ 82 years, only AFB-positive with positive culture results were evident (2/13). Of all AFB-negative with positive culture cases, only two patients had a history of previous treatment (2/167).

## 4 Discussion

This study evaluated the confirmatory culture of TB following the AFB test, demonstrating moderate performance of the sputum smear in Peruvian patients aged ≥ 65 years. Due to the high false-negative rate and low sensitivity of AFB, particularly in screening for EPTB, performing a culture is crucial to avoid diagnostic errors in elderly patients. Also, the rate of negative sputum smears with positive cultures was slightly higher in patients aged ≥ 82 years.

The performance of the AFB test was assessed based on diagnostic agreement and the rate of contaminated cultures. During PTB diagnosis, a high specificity for the AFB test was observed in sputum samples. Similarly, high specificity was noted in extrapulmonary samples. However, the diagnostic sensitivity was lower than 79% for PTB and less than 50% for EPTB screening, highlighting important limitations of this diagnostic test in primary healthcare settings (Sulis et al., 2016). Previous studies have reported different levels of diagnostic performance for the AFB test (Symes et al., 2020; Park et al., 2014; Cruz-Hervert et al., 2012; Monkongdee et al., 2009; Grupo de trabajo de expertos en tuberculosis, and Grupo de trabajo de Comunidades Autónomas, 2009). Its relevance is particularly evident in the diagnostic assessment of patients with previous treatment, where culture alone should not be the sole useful method for evaluating treatment efficacy (Sharma et al., 2022; Symes et al., 2020; Grupo de trabajo de expertos en tuberculosis, and Grupo de trabajo de Comunidades Autónomas, 2009; Caulfield and Wengenack, 2016).

The diagnostic concordance between AFB and culture was very low, with only 1.1% of sputum smears in pulmonary samples and 0.05% of extrapulmonary samples showed concordance. Despite the limited number of tests with available data, these showed moderate to very good agreement (Figures 2, 3). This evaluation is influenced by multiple factors including specimen collection, the analytical process, the type of diagnostic method used, and the skill and specialization of health workers responsible for laboratory diagnosis (Teo et al., 2020; Ssengooba et al., 2012; Aber et al., 1980). It is important to consider elderly patients with previous treatment as they could skew the performance of the AFB smear test. However, none of the patients with EPTB analyzed had previous treatment, and for PTB, the two patients with previous treatment were not statistically significant. Therefore, our performance data are robust and free from biases that could hinder their interpretation.

Culturing *Mycobacterium tuberculosis* is essential as it provides a higher diagnostic yield, allows for the identification of the causative agent, and facilitates antimicrobial susceptibility testing. Several studies have demonstrated the high performance of Ogawa-Kudoh agar for isolating TB in sputum samples, significantly contributing to routine diagnostic processes (Rivas et al., 2010; Silva et al., 2013; Agapito et al., 2009; Palaci et al., 2013). However, the performance of this method depends on various factors such as the type of egg used, the type of sample cultivated, and the decontamination time of the samples (Barrera, 2008; Agapito et al., 2009; Rivas et al., 2010; Asencios et al., 2012; Silva et al., 2013; Palaci et al., 2013; Singhal et al., 2014; Noori et al., 2015).

Another factor that may influence performance is the composition of the medium (Rivas et al., 2010; Sequeira de Latini and Barrera, 2008; Barrera, 2008). Ogawa medium uses a base of dried egg and potato starch, whereas Lowenstein-Jensen medium is based on whole egg, glycerol, and mineral salts (Kim and Ryoo, 2011; Lubasi et al., 2004). Ogawa is quicker to prepare and less susceptible to contamination, while Lowenstein-Jensen provides a richer environment for bacterial growth (Ariami et al., 2018). Large-scale performance comparisons of these media are needed to determine which is better for elderly samples, which may be contaminated by hospital procedures (i.e., endotracheal intubation), have altered sample composition affecting culture outcomes, and may contain contaminants.

Furthermore, we investigated the rate of contaminated cultures in PTB screening and found that 14.2% presented contamination. This contamination rate is more than two times higher reported in previous studies, which range from 5.5% to 7% (Yvette et al., 1995; Rivas et al., 2010). Nonetheless, Ogawa agar has demonstrated a high diagnostic yield with a lower rate of contaminated cultures compared to the Lowenstein-Jensen medium (Ceyhan et al., 2012), using the N-acetyl-L-cysteine-sodium hydroxide decontamination technique (Jobarteh et al., 2017), and with modified Ogawa medium (Lubasi et al., 2004).

Extrapulmonary samples, especially stool samples, typically have higher contamination rates than pulmonary samples. However, in our study, the most frequent contaminated cultures were bronchial aspirate (4.6%), gastric aspirate (3.8%), tracheal aspirate (1.7%), and urine (1.1%). These results differ from previous reports and may explain the low frequency of contaminated cultures in comparison to sputum cultures (Symes et al., 2020; Houston and Macallan, 2014; Banta et al., 2020).

The lack of confirmation of TB cultivation in many studies may account for the differing findings related to the proportion of TB cases in the elderly compared to younger individuals (Sood, 2000; Cruz-Hervert et al., 2012; Shivam et al., 2014; Azadi et al., 2018). Although a lower frequency of positive smears has been reported in patients ≥ 65 years, our findings indicate a higher frequency of positive smears in patients aged 65 to 81 years compared to those older than 81 (Table 1).

In the other hand, another critical finding to emphasize is the proportion of false negatives observed in sputum smears (21.8%) and extrapulmonary samples (54.5%). As previously mentioned, the clinical presentation of TB in the elderly is highly heterogeneous. Assuming that over 20% of samples evaluated in these patients contain errors, the results could lead to loss of follow-up and control, and a resurgence of the disease in this population, which already faces high physiological, immunological, nutritional, and social risk factors (Sood, 2000; Yoshikawa and Rajagopalan, 2001; Cruz-Hervert et al., 2012; Zagaria, 2008; Moya-Salazar et al., 2018).

The analysis by age group revealed that the rate of sputum smear-negative with positive culture was significantly higher in patients aged ≥ 82 years with PTB. The concerning proportion of false negatives (20.3%) in this age group could lead to diagnostic errors, exposing patients to unnecessary risks, such as invasive investigations or loss of follow-up, thereby compromising prevention programs. This is particularly dangerous given the high comorbidity in the elderly (Schaaf et al., 2010; Salive, 2013). Recently, in Peru, it has been reported that 11–20% of TB cases occur in people ≥ 65 years, primarily men (Alarcón et al., 2017; Ministerio de Salud, 2024). For these patients, new diagnostic and therapeutic methods need to be evaluated according to social realities and age groups to ensure efficient management of older adults with TB (Lienhardt et al., 2016; Moya-Salazar et al., 2018).

Additionally, our diagnostic safety analysis revealed that the quality of results was suboptimal. For instance, the diagnosis of EPTB yielded only half the ideal value (Youden’s J = 0.5). Due to the poor quality of samples and the challenges in obtaining and analyzing them solely with AFB, culture should be the cornerstone of the TB diagnosis algorithm in older patients. However, expanded testing by smear culture is recommended but challenging to implement in remote and impoverished areas. A more feasible method might be antibody serology, which has shown previous validation (Ivanyi, 2012; Bothamley et al., 1992). Other techniques include liquid culture and fluorescence analysis but are expensive for poor settings (). These need evaluation in a post-pandemic context, as COVID-19 has impacted the development of many diseases and shifted healthcare priorities (Robles et al., 2024; Moya-Salazar et al., 2024; Dutta et al., 2023; Khunti et al., 2022, World Health Organization, 2021). Despite this, governments have continued TB control programs worldwide (Ministerio de Salud, 2024; Williams et al., 2023). It is now crucial to leverage pandemic-era technological advances to enhance TB surveillance and ensure equitable access to care.

This study had limitations. (i) A larger sample size of older patients with EPTB should be evaluated to fully understand their clinical characteristics and their diagnostic patterns. Furthermore, it is crucial to evaluate whether patients over 81 years old are clinically different from those under 81 years old, as well as to compare these results with younger populations to identify differences in the development of AFB and culture for PTB and EPTB. (ii) The impact of the contaminated culture should be estimated due to processing errors. The impact of contaminated cultures should be estimated due to processing errors. In this study, 4.3% of contaminated cultures had positive sputum smears, creating uncertainty about the results and potentially affecting the prevention and treatment of patients with EPTB. (iii) Given Peru’s high rates of MDR-TB (World Health Organization, 2022), similar studies should be conducted to assess the efficacy of diagnostic procedures for resistant isolates (MDR- and XDR-TB). In this study, such cases were excluded to avoid bias; however, their influence on performance, as previously reported in the adult population (Tanoue et al., 2002), should not be overlooked. (iv) We found that the older the patient, the higher the proportion of negative AFB smears. Multicenter and multiregional studies should be conducted in populations with similar TB incidence rates to accurately estimate AFB results and determine the need for culture in low-income primary health-care centers (Mamuye, 2012). Finally, (v) Other diagnostic methods, such as molecular tests and the Lowenstein-Jensen culture medium, should be compared to identify the most optimal diagnostic tests for elderly patients with PTB and EPTB (Thomas and Rajagopalan, 2001; Schaaf et al., 2010). Additionally, it is important to report a list of contaminants and disclose any specific microbial associations or mixtures responsible for the contamination of diagnostic samples.

## 5 Conclusions and future directions

The evaluation of confirmatory TB culture in pulmonary and extrapulmonary samples after AFB smear testing in elderly patients is essential due to the moderate performance of AFB, particularly in patients ≥ 82 years and in sputum samples. Diagnosing tuberculosis in the elderly poses a significant challenge for health systems globally, especially in regions where TB remains almost endemic. Recognizing the limitations of current diagnostic tests is crucial to proposing improvements in diagnostic processes within the framework of the global zero TB plan.

Future studies should investigate how the timing and schedule of TB treatment may influence the results of bacteriological tests. Additionally, analyzing the risk of infection transmission by patients with smear-negative and culture-positive pulmonary TB is imperative. These specific aspects of TB in the elderly may be key pieces in the puzzle of achieving successful diagnosis and effective treatment.

## Data Availability

The original contributions presented in the study are included in the article/supplementary material, further inquiries can be directed to the corresponding author.
